# Pericardial tamponade during surgical ventriculoperitoneal shunt placement : a case report and review of the literature

**DOI:** 10.1186/s12871-026-03612-6

**Published:** 2026-01-15

**Authors:** Bin-hao Ruan, Li Chen, Dong-hang Cao, Na-na Wang

**Affiliations:** 1 Taizhou Hospital of Zhejiang Province, Taizhou, Zhejiang China; 2 Eenze Hospital, Taizhou, Zhejiang China

**Keywords:** Complications, Pericardial tamponade, Ventriculoperitoneal shunt, Case report, Echocardiography

## Abstract

**Background:**

Cardiac tamponade is a catastrophic hemodynamic emergency. While ventriculoperitoneal (VP) shunt placement is a common neurosurgical procedure, its typical complications include obstruction and infection. No prior cases of intraoperative cardiac tamponade during VP shunt placement have been reported.

**Case presentation:**

We present a case of life-threatening cardiac tamponade that occurred during VP shunt insertion in a patient with severe cerebral edema. The patient developed abrupt hemodynamic collapse, progressing to pulseless electrical activity requiring emergency cardiopulmonary resuscitation.

**Conclusions:**

Prompt diagnosis was achieved by point-of-care ultrasonography, and immediate pericardiocentesis was life-saving. This case illustrates a previously unreported, lethal complication of VP shunt surgery, potentially resulting from unintended cardiac penetration by the ventricular catheter. We alert anesthesiologists and neurosurgeons to consider this etiology in cases of unexplained intraoperative collapse and advocate for the ready availability of ultrasound for rapid diagnosis.

Ventriculoperitoneal (VP) shunting is a cornerstone neurosurgical procedure for the management of hydrocephalus. The technique involves the implantation of a catheter system, with the proximal end placed in the cerebral ventricle and the distal end tunneled subcutaneously to the peritoneal cavity. An adjustable valve, typically integrated into the system, regulates cerebrospinal fluid (CSF) drainage based on intracranial pressure (ICP), thereby achieving therapeutic control of hydrocephalus. Established complications of VP shunts primarily include obstruction and infection (e.g., meningitis or ventriculitis) [1–3]. In contrast, pericardial tamponade represents an exceptionally rare and previously unreported complication of this procedure. This case report aims to heighten awareness among anesthesiologists regarding this potential intraoperative catastrophe. We emphasize the critical role of point-of-care ultrasound (POCUS) for the rapid diagnosis of pericardial tamponade in the context of unexplained hemodynamic instability, enabling immediate life-saving intervention and ensuring patient safety.

## Case introduction

The patient is a 66-year-old male farmer with a history of hospitalization over eight months ago for a fall from height. A cranial CT scan revealed hemorrhagic lesions along the cerebral falx and tentorium cerebelli, a right frontotemporal parietal occipital intracerebral hemorrhage. He underwent emergency surgical evacuation of the right intracerebral hematoma with decompressive craniectomy. A delayed intracerebral hematoma was identified on second day, so a second procedure for the left-sided hematoma. Following these interventions, the patient was transferred to a rehabilitation facility. During his rehabilitation course, he experienced recurrent episodes of unresponsiveness accompanied by generalized tonic-clonic seizures. Subsequent imaging confirmed the development of hydrocephalus, leading to the current admission for planned placement of a lateral ventriculoperitoneal shunt.

Preoperative evaluation included an ECG, which demonstrated sinus rhythm with ventricular premature complexes and an incomplete right bundle branch block. Ehocardiography showed ventricular septal thickening and impaired left ventricular diastolic function. Chest CT imaging indicated bilateral pneumonia with segmental atelectasis and evidence of old bilateral rib fractures. Pertinent laboratory findings were as follows: hemoglobin 11.6 g/dL, albumin 34.6 g/L, high-sensitivity troponin T 0.033 ng/mL, pro-BNP 147 pg/mL, and C-reactive protein 14.5 mg/L. Arterial blood gas analysis revealed an oxygenation index of 660 mmHg. Complete blood count, thyroid function, and coagulation profiles were within normal limits.

The patient was admitted to the operating room with his eyes open but in a delirious state. He had a pre-existing tracheostomy and was breathing spontaneously. Invasive arterial pressure monitoring was established following anesthesia induction. Hypotension occurred post-induction but responded to intravenous ephedrine (6 mg), accelerated fluid administration, and initiation of a norepinephrine infusion (0.03 µg/kg/min). Subsequently, his vital signs stabilized, with blood pressure maintained at approximately 120/60 mmHg.

**Fig. 1:**
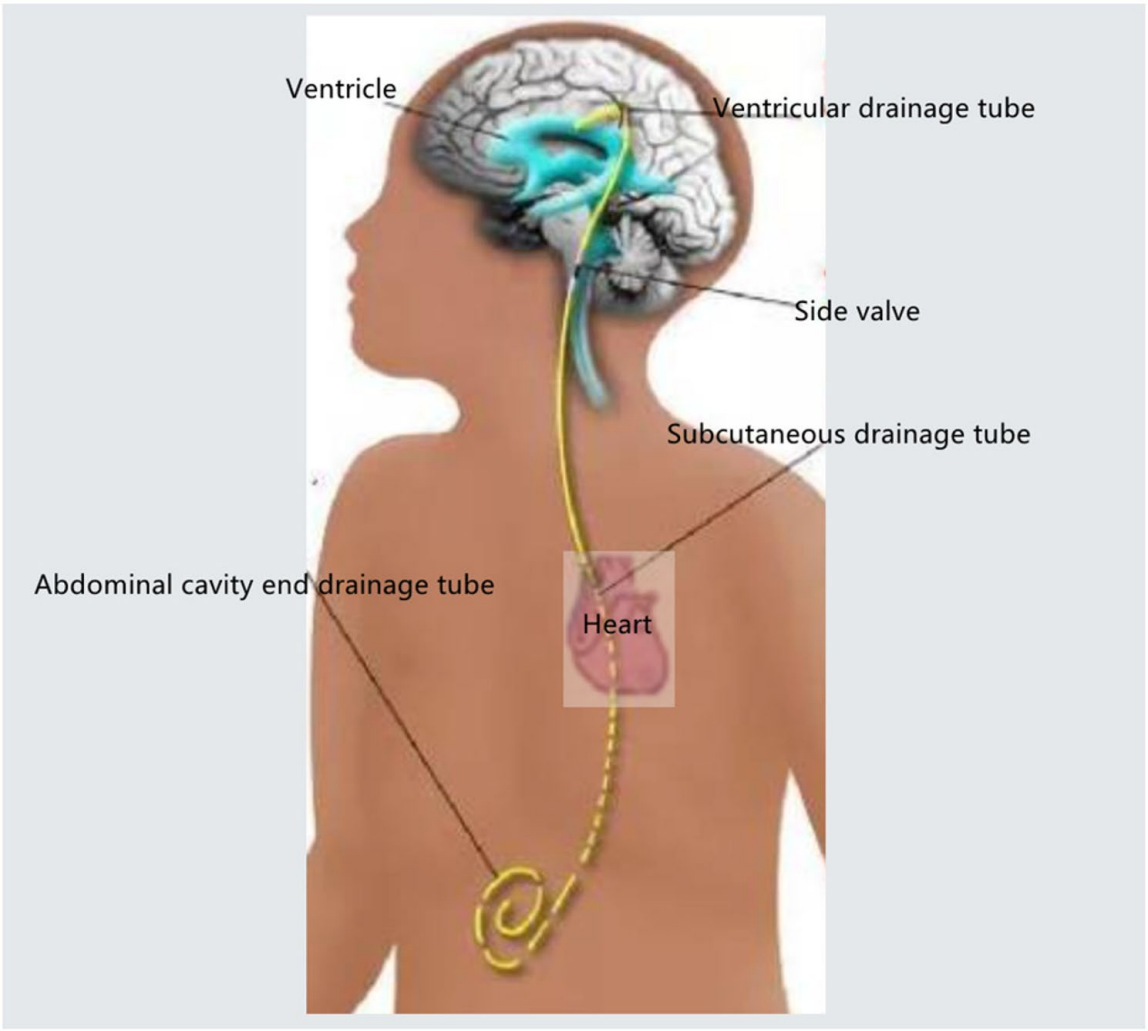
The route diagram of the drainage tube, passing through the subcutaneous tissue above the heart

**Fig. 2:**
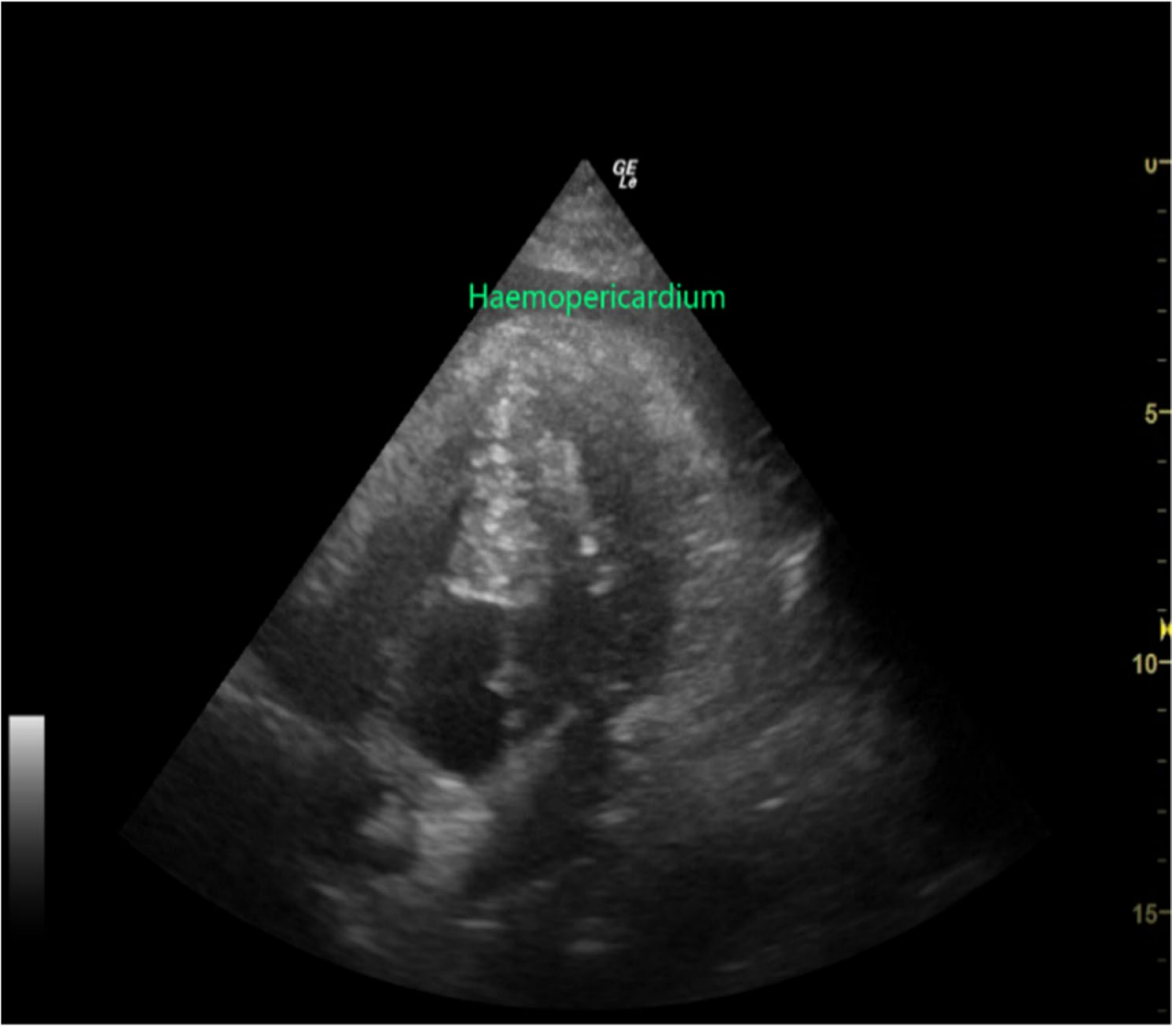
The intraoperative echocardiography revealed significant pericardial effusion

The surgery was performed on the left side due to altered cerebral anatomy on the right from previous operations. The surgical procedure involved drilling a burr hole at the scalp incision site, inserting the ventricular catheter and valve into the lateral ventricle, and then creating a subcutaneous tunnel. The abdominal catheter was passed through this tunnel from the cranial incision to the abdominal site, traversing the left parietotemporal region, retroauricular area, occipital region, and chest wall (Fig. 1). Following tunnel creation, the catheter tip was positioned under laparoscopic guidance. Approximately 90 min into the procedure, the anesthesiologist noted a sudden decrease in the patient’s blood pressure, with invasive arterial pressure dropping to approximately 60/32 mmHg.An immediate call for assistance was made, and intermittent boluses of norepinephrine (60 µg), ephedrine (6 mg), and epinephrine (10 µg) were administered. The blood pressure transiently rose but decreased again after about two minutes. Repeated epinephrine boluses resulted only in brief hypertensive episodes followed by rapid deterioration. Communication with the surgeon confirmed no active bleeding upon careful laparoscopic exploration of the abdominal cavity. To rule out an allergic reaction to hydroxyethyl starch, the infusion was switched to lactated Ringer’s solution, and intravenous hydrocortisone (100 mg) was administered. Arterial blood gas analysis revealed a partial pressure of oxygen (PaO₂) of 463 mmHg. Auscultation of both lungs was clear, and airway pressures were not elevated, making pneumothorax an unlikely cause. At this time, it became difficult to increase the blood pressure of the patient, the heart rate continued to drop, the blood pressure quickly decreased to 30 mmHg and the heart rate quickly decreased to 10 beats/min. Immediately CPR was performed. After 2–3 min, the heart rate recovered to 130 beats/min. An immediate echocardiogram of the heart revealed a significant pericardial effusion(Fig. 2).

A multidisciplinary consultation was urgently obtained from the departments of Thoracic Surgery, Radiology, and others. Emergent pericardiocentesis was performed under ultrasound guidance, with the drainage of approximately 100 ml of serosanguinous fluid. This intervention resulted in the immediate stabilization of the patient’s hemodynamic status. Post-procedure, the patient was transferred to the intensive care unit (ICU) and administered tranexamic acid (0.5 g once daily) for hemostasis.

The postoperative course was monitored closely. The pericardial drainage volume was 240 ml on the first postoperative day and 183 ml on the second. The pericardial drain was ultimately removed on the 15th postoperative day, and the patient was discharged one month after the surgery.

**Fig. 3:**
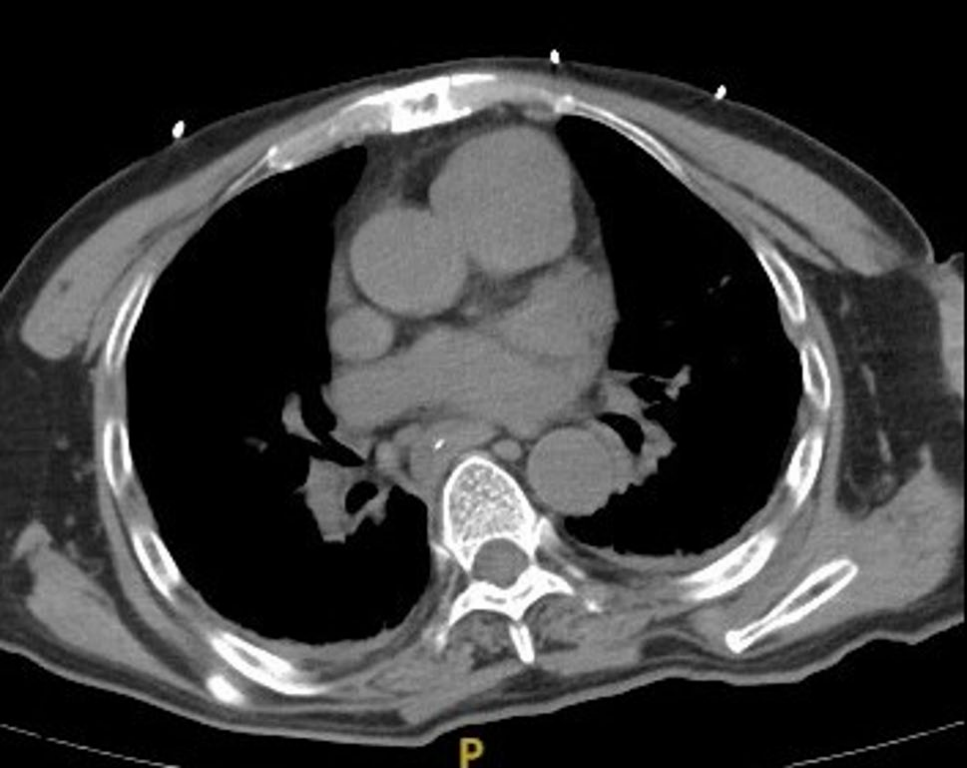
The preoperative CT showed no internal or external pericardial effusion

**Fig. 4:**
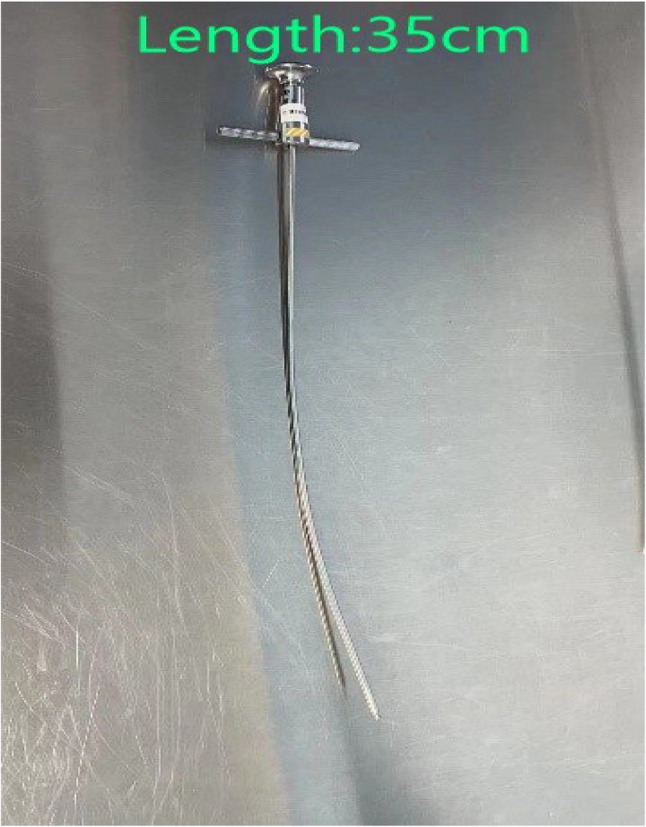
The postoperative CT reveals blood accumulation between the pericardium and the ribs and indicates the possibility of intraoperative injury. The white arrow indicates the position of the catheter. The black arrow indicates the location of the accumulated blood

A hospital-wide multidisciplinary discussion was convened on the second postoperative day to investigate the etiology of the tamponade. The attending neurosurgeon postulated that pre-existing adhesions from a prior rib fracture history, combined with repeated subcutaneous tunneling, might have led to traction-induced pericardial vessel injury. The thoracic surgeon conducted a detailed comparative analysis of pre- and postoperative computed tomography (CT) scans. The postoperative CT (Fig. 3) revealed a hematoma between the pericardium and the sternum, which was not present on the preoperative imaging. This finding led to the conclusion that the injury was iatrogenic, occurring during the surgical procedure. A retrospective review of the anesthetic record provided further evidence, showing a marked decrease in blood pressure approximately 3–5 min after the surgeon began creating the subcutaneous tunnel from the neck to the chest. This temporal correlation strongly supports the mechanism of iatrogenic vascular injury causing hemopericardium (Fig. 4).

## Discussion

Ventriculoperitoneal (VP) shunt placement is a standard neurosurgical procedure for the management of hydrocephalus. While generally considered safe, it is associated with well-documented complications, predominantly infection and, less commonly, pneumothorax. However, to the best of our knowledge, no prior cases of intraoperative pericardial tamponade directly attributable to the shunt insertion procedure itself have been reported in the literature. The diagnosis of this complication can be challenging, even for experienced surgeons. In the present case, the operating surgeon was an associate chief physician with substantial clinical experience and a history of numerous successfully performed VP shunt procedures. When the patient developed acute circulatory instability, the initial differential diagnosis, understandably, included pneumothorax, but did not initially consider pericardial tamponade. This highlights the diagnostic dilemma posed by this rare event. Cardiac tamponade is a life-threatening condition characterized by the accumulation of fluid in the pericardial space, leading to impaired cardiac filling, a precipitous drop in cardiac output, and circulatory shock [4]. Iatrogenic injury is a significant cause, with one study identifying it as the most prevalent etiology (36%), primarily resulting from percutaneous cardiac interventions [5]. Beyond cardiology, tamponade has been reported as a rare complication in other surgical disciplines. For instance, eight cases have been documented during lung lobe resection, with a predilection for left-sided procedures, and proposed mechanisms include vascular anomalies, coronary artery injury, or stapler malfunction [6–13]. A case during partial hepatectomy was attributed to an incomplete inferior vena cava suture, allowing blood to track into the pericardial cavity [14].

**Fig. 5:**
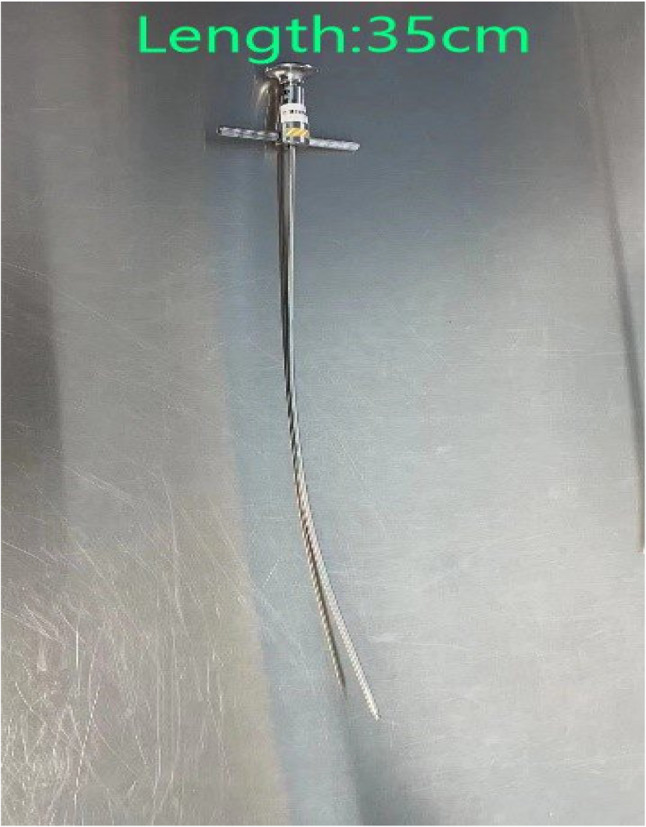
Subcutaneous tunnel reaming device

It is crucial to distinguish the present case from a previously reported case of tamponade following VP shunt surgery. That prior instance, occurring in a two-month-old infant, was caused by delayed migration of the distal catheter from the peritoneal cavity into the pericardial space [15]. In contrast, our case involved an acute intraoperative hemopericardium due to direct vascular injury during the subcutaneous tunneling phase, a mechanism that has not been previously described. Furthermore, the diagnostic challenge is compounded by the potential for delayed symptom onset, which may occur after the primary surgical focus has shifted, thereby delaying recognition (Fig. 5).

## Conclusions

This case reminds us that the possibility of pericardial tamponade should be considered when hemodynamic instability, refractory hypotension, and unexplained cardiac arrest occur in the surgery across the cardiac region.

## Data Availability

Data sharing is not applicable to this article as no datasets were generated or analysed during the current study.
